# Therapeutic effects of histone deacetylase inhibitors in a murine asthma model

**DOI:** 10.1007/s00011-016-0984-4

**Published:** 2016-08-26

**Authors:** Yuan Ren, Xinming Su, Lingfei Kong, Menglu Li, Xuan Zhao, Na Yu, Jian Kang

**Affiliations:** Department of Respiratory Medicine, Institute of Respiratory Diseases, The First Affiliated Hospital of China Medical University, 155 North Nanjing Street, Shenyang, 110001 People’s Republic of China

**Keywords:** Bronchial asthma, Histone deacetylase, Airway inflammation, Airway hyperresponsiveness

## Abstract

**Objective and design:**

To investigate the therapeutic effects of various HDAC inhibitors on the development of chronic allergic airway disease in mice with airway inflammation, airway remodeling, and airway hyperresponsiveness.

**Subjects:**

Wild-type BALB/C mice (*N* = 72).

**Treatment:**

Tubastatin A HCl [TSA, a selective histone deacetylase 6 (HDAC6) inhibitor], PCI-34051 (a selective HDAC8 inhibitor), and givinostat (a broad-spectrum HDAC inhibitor that inhibits class I and class II HDACs and several pro-inflammatory cytokines).

**Methods:**

Mice were divided into six groups: control, asthma, dexamethasone (positive control), TSA, PCI-34051, and givinostat (*n* = 12 per group). Twenty-four hours after OVA nebulization, airway hyperresponsiveness, inflammation, and remodeling were assessed.

**Results:**

The chronic asthma mouse model produced typical airway inflammation, airway remodeling, and airway hyperresponsiveness. Administration of PCI-34051 and dexamethasone reduced the eosinophilic inflammation and airway hyperresponsiveness in asthma to reduce the airway remodeling. Treatment with Tubastatin A HCl reduced airway inflammation and was associated with decreased IL-4, IL-5 and total inflammatory cell count, as well as goblet cell metaplasia and subepithelial fibrosis; however, this outcome was not as effective as that with dexamethasone. TGF-β1 expression in the cytoplasm of airway epithelium of mice in the Tubastatin A HCl group was reduced and expression of α-SMA in the airway smooth muscle was also decreased.

**Conclusions:**

The results suggested that treatment with HDAC inhibitors can reduce airway inflammation, airway remodeling, and airway hyperresponsiveness in chronic allergic airway disease in mice.

## Introduction

Asthma is a chronic respiratory disease having pathological features such as airway inflammation, airway remodeling and airway hyperresponsiveness. Long-term repeated asthma attacks may induce thickening of the bronchial airway smooth muscle, subepithelial fibrosis, and thickening of the basement membrane resulting in airway remodeling and airway collapse [1]. Currently, corticosteroids and long-acting β2-agonists are the mainstays of asthma therapies; however, they do not directly target airway remodeling (structural changes to the airway wall, including fibrosis) or airway epithelial susceptibility and damage [2].

The development of anti-inflammatory drugs that could complement the currently available therapeutic modalities is ongoing. Histone deacetylase inhibitors are one such group of compounds. Developments have been made in the understanding of histone deacetylase expression in normal and diseased airways and pulmonary tissue, as well as effects of histone deacetylase inhibitors on structural and inflammatory cells in the lung, including cell proliferation, differentiation, and apoptosis and senescence. It has been demonstrated that histone deacetylase inhibitors not only inhibit tumor cell proliferation, but also have anti-inflammatory, anti-fibrotic and anti-angiogenic qualities [1–6], which can be used for the treatment of airway remodeling in patients with asthma. Cell experiments and animal experiments have verified that histone acetylation enzyme inhibitors can resist cell proliferation, angiogenesis, oxidation, inflammation and fibrosis [3–7].

Selective inhibition of HDAC6 can promote hyperacetylation of α-tubulin and HSP90 to inhibit cell motility and accelerate degradation of HSP90 client proteins, while it has no significant effect on normal cells [8–12]. This may be the main reason of HDAC6 inhibitors resisting metastasis and angiogenesis. Histone acetylation enzymes 8 (HDAC8) is expressed in the heart, lungs, liver and kidneys [7], is an essential factor in the cell survival and proliferation [8], and it also can regulate differentiation and contraction of the smooth muscles. Therefore, application of HDAC8-specific inhibitors may represent a mode of treatment for asthma [9, 10].

In the current study, the effect of an HDAC6 inhibitor (Tubastatin A HCl) and an HDAC8 inhibitor (PCI-34051), on airway inflammation, remodeling, and hyperresponsiveness in a mouse model of chronic asthma was investigated.

## Materials and methods

### Mice and treatment

A mouse model of asthma was utilized as previously described [13, 14]. Briefly, healthy female BALB/C mice (*n* = 72) aged 6–8 weeks and weighing 18–22 g were purchased from the Liaoning Changsheng Biotech Co., Ltd (license no: SCXK[liao]2010-0001). Animals were housed independently in a pathogen-free room and provided ad libitum access to water and standard food. Animals were housed for 1 week prior to experiment onset. Mice were divided into six treatment groups: normal control, simple asthma, dexamethasone, Tubastatin A HCl, PCI-34051, and givinostat. Dexamethasone was purchased from Zhuo Feng Pharmaceutical Co., Ltd., Zhengzhou, China. All HDAC inhibitors were purchased from Selleckchem, Houston, TX, USA. Sensitization was carried out for mice in the last five groups on the 1st, 8th and 15th day using ovalbumin (OVA, 20 μg; Sigma, St. Louis, MO, USA) and aluminum hydroxide gel (2 mg; Sigma). 7 days after the last sensitization, OVA (20 mg/ml) atomization was performed using an ultrasonic atomizing device (3 ml/min for 30 min, 3 times/week for 8 weeks). Dexamethasone (2.0 mg/kg) [15], TSA (0.5 mg/kg) [16], PCI-34051 (0.5 mg/kg) and givinostat (0.5 mg/kg) were administered via intraperitoneal injection 30 min before excitation. In the normal control group, normal saline was used instead of OVA. All procedures were approved by the institutional animal care and use committee (IACUC) at The First Affiliated Hospital of China Medical University, Republic of China.

### Detection of airway responsiveness

As previously described [17], airway resistance was measured 24 h following excitation using non-invasive whole body plethysmography to test pulmonary function (Emka Technologies, Paris, France). Enhanced pause (Penh) was recorded 3 min following administration of acetyl-β-methylcholine chloride at concentrations of 0, 3.125, 6.25, 12.5, 25, and 50 mg/ml (Sigma) to detect airway resistance.

### Detection of cytokines in the bronchoalveolar lavage fluid using ELISA

Bronchoalveolar lavage fluid was centrifuged at 1200 r/min for 5 min. Supernatant was collected and stored at −80 °C. Precipitated cells were resuspended in 0.4 ml Hank’s Balanced Salt Solution (HBSS) and 0.1 ml was taken for the total cell count. Remaining cells were smeared onto a clean slide and a differential count was performed after Wright–Giemsa staining. IL-4, IL-5, IFN-γ, and TGF-β1 levels in the bronchoalveolar lavage fluid (BALF) were measured using ELISA assay kits (CUSABIO, Hubei, China) per manufacturer’s instructions.

### Hematoxylin and eosin staining

Paraffin-embedded tissue sections were dewaxed, stained with hematoxylin (5–10 min), washed in running tap water (5 min), differentiated with hydrochloric acid at room temperature (3 s), washed in running tap water (30 min), stained with eosin (1–2 min), washed in running tap water (1 min), dehydrated in a graded alcohol series, vitrificated in xylene, and mounted in resinous medium. The inflammatory changes of the lung tissue harvested from various groups were observed using a light microscope.

### AB-PAS staining

The paraffin-embedded tissue section was dewaxed, washed in 3 % acetic acid, stained with Alcian blue (Leagene, Beijing, China), washed in distilled water, stained with 0.5 % periodic acid, washed in distilled water, washed in 70 % alcohol, stained with Schiff’s reagent (15 min), washed in running tap water (10 min), stained with hematoxylin, washed in running tap water, dehydrated using a graded alcohol series, vitrificated in xylene, and mounted in resinous medium. The proliferation of goblet cells in the airway was observed using a light microscope.

### Masson’s trichrome staining

The paraffin-embedded tissue section was dewaxed, stained with Masson’s composite staining solution (Fuzhou Maixin Bio, Fujian, China) for 5 min, washed in 0.2 % acetic acid, stained with 5 % tungsten molybdate (5–10 min), washed in 0.2 % acetic acid twice, stained with aniline blue (5 min), dehydrated in a graded alcohol series, vitrificated in xylene, and mounted in resinous medium. The subepithelial collagen deposition was observed using a light microscope.

### Quantification of various types of cells in BALF

The counting of total cells and cells of different types in BALF was performed using a light microscope with a randomly selected visual field (200 cells were counted in each field). These cells included macrophages (MC), eosinophils (EOS), neutrophils (N) and lymphocytes (LC). The percentages of cell types were determined by counting the number of cells of a specific type per 200 cells.

### Detection of α-SMA and TGF-β1 expression using semi-quantitative immunohistochemistry

Paraffin-embedded tissue sections were de-paraffinized, hydrated, and washed in PBS three times (5 min each). Each section was then incubated in 3 % H_2_O_2_ at room temperature and washed in PBS three times (5 min each). Sections were then treated with 0.01 M citrate buffer (pH 6.0) for antigen retrieval in a microwave oven four times (6 min for each). Sections were then blocked with 15 % goat serum at room temperature for 30 min, and then incubated with primary antibody (1:1000) at 4 °C overnight. The primary antibodies included anti-TGF-β1 antibody (Abcam, Cambridge, UK) anti-alpha smooth muscle actin antibody (Abcam), while the secondary antibody (1:500 at room temperature for 60 min) was Peroxidase-Conjugated AffiniPure IgG (Zhongshan Golden Bridge, Beijing, China). After PBS wash, sections were placed in the DAB color development solution (5–10 min), rinsed sufficiently in running tap water, counterstained, dehydrated, vitrificated, and mounted.

### Measurement of α-SMA and TGF-β1 expression in the lung tissue

Protein expression of α-SMA and TGF-β1 was measured using Western blot assay. Briefly, SDS polyacrylamide gel electrophoresis (20 μg total protein, pre-stained with 4 μl marker, treated with 10 % separating gel for 30 min at 80 V, followed by 90 min of electrophoretic separation), followed by sample incubation with the primary antibodies [α-SMA (Abcam) and TGF-β1 (Abcam), 1:1000]. A highly sensitive chemiluminescent detection kit (Super ECL Plus; PPLYGEN, Beijing, China) was used to measure protein expression. Sample imaging was carried out using the UVP imaging system (Upland, CA, USA).

### Statistical analysis

Data were presented as mean ± standard deviation (SD) for each group. Baseline Penh values and other outcomes were compared using a one-way ANOVA with a post hoc pair-wise comparison (Bonferroni test). The Penh value for a given concentration of methacholine (MCh) among groups was compared using a two-way ANOVA adjusting baseline Penh value with a post hoc pair-wise comparison (Bonferroni test). Furthermore, the one-sample test was performed to compare the expression of western blot with that of normal control group (setting mean = 1). The differences among the remaining five groups were compared using a one-way ANOVA with a post hoc pair-wise comparison (Bonferroni test). All statistical assessments were two-tailed and considered significantly different as *p* < 0.05. All statistical analyses were carried out using IBM SPSS statistical software version 22 for Windows (IBM Corp., Armonk, NY, USA).

## Results

The chronic asthma model elicited the expected physiological outcomes. OVA-excited mice in the asthma group demonstrated sneezing, nasal itching, catching the ear, urinary and fecal incontinence, and asthma. Furthermore, the body weight of mice 8 weeks after atomization was reduced significantly, mental state was poor, the mice became less active, and their hair lost its shine. These symptoms were ameliorated in the asthma group, the dexamethasone group, TSA HCl group, PCI-34051 group, and givinostat groups. In the normal group, mice did not experience asthma attacks.

**Fig. 1:**
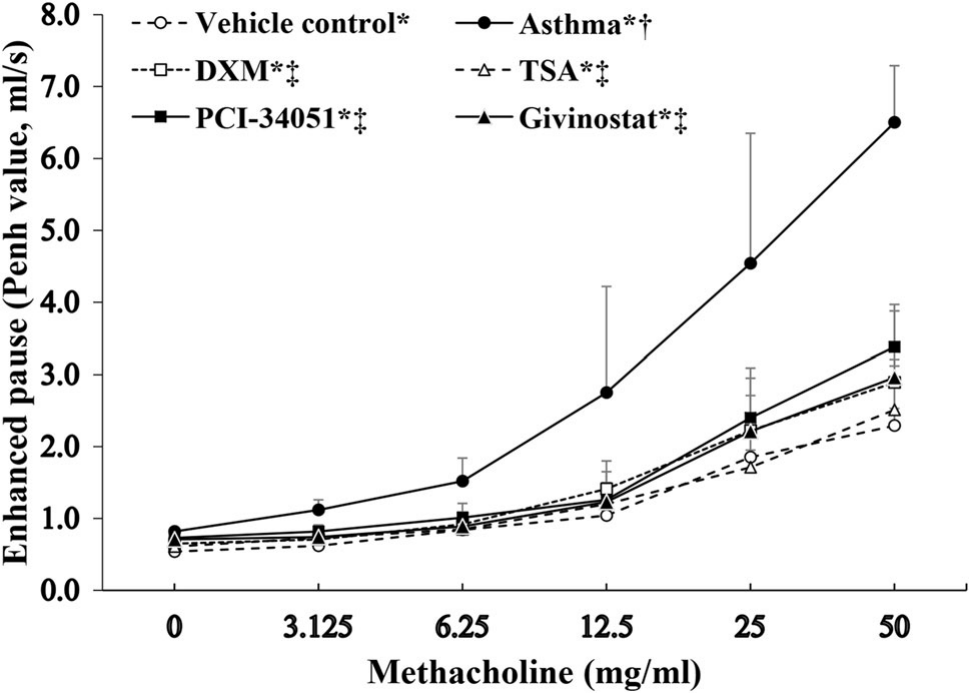
Enhanced pause (Penh values) following stimulation with acetyl-β-methylcholine chloride (MCh). Data presented as mean ± SD for each MCh group (*n* = 8 for each group). Differences in Penh value change over concentration of MCh among groups were compared using a two-way ANOVA adjusted for baseline Penh values. *p < 0.05, indicated the Penh values was increased along with the increasing over various concentrations of MCh for each group (all *p* values < 0.001). *p* < 0.05, compared with ^†^normal control group, or ^‡^asthma group

The Penh values for all groups were increased with the increased concentration of Acetyl-β-methylcholine chloride (all *p* < 0.05; Fig. 1). MCh was associated with significantly greater Penh values in the asthma group and PCI-34051 group compared with the normal control group. All four treatment groups, including DXM, TSA, PCI-34051, and givinostat groups, had significantly lower Penh values compared with the asthma group. The TSA group had significantly lower Penh values compared with the DXM group (all *p* < 0.05). There were no significant differences between TSA, PCI-34051, and givinostat groups (Fig. 1).

**Fig. 2:**
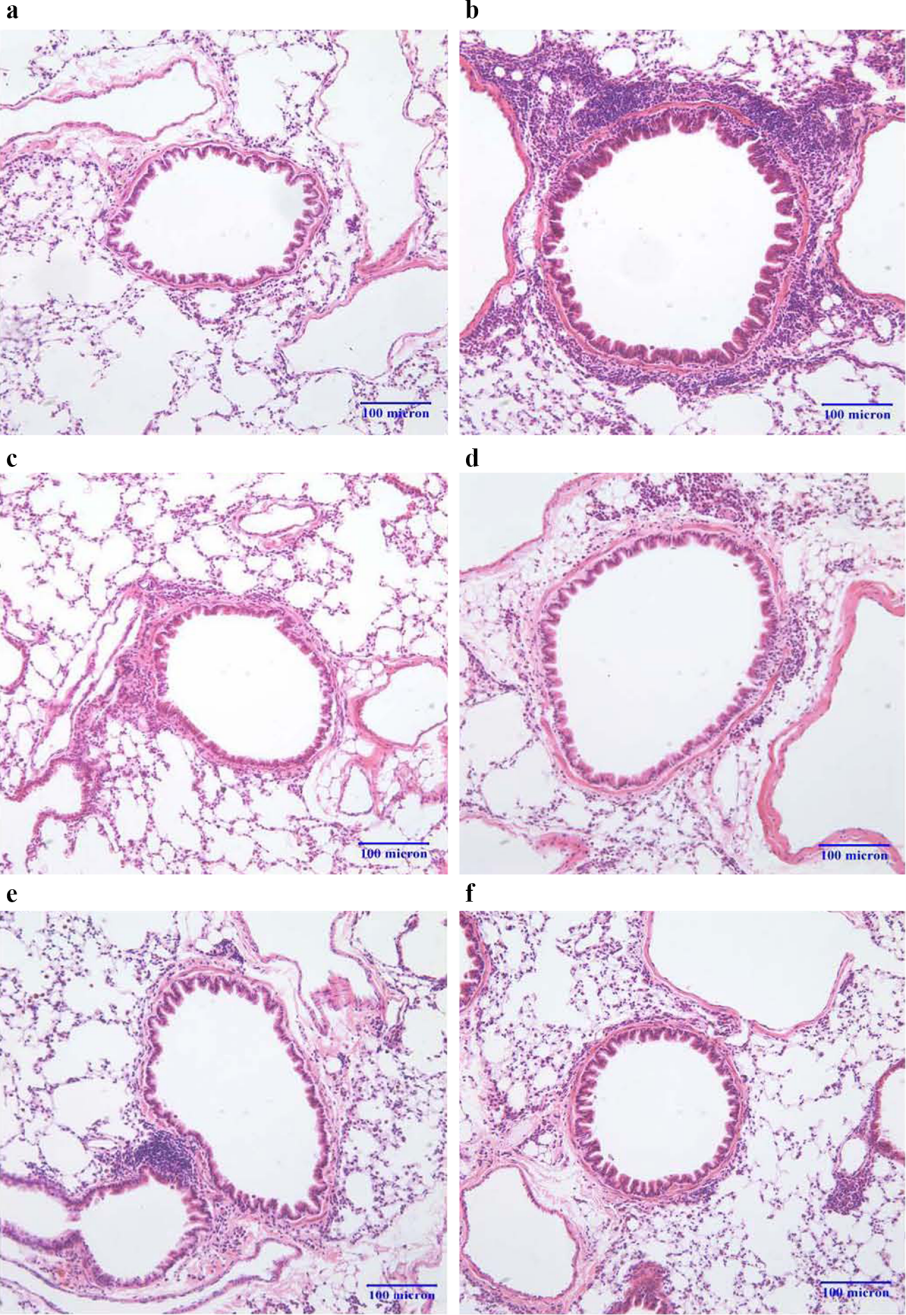
Representative lung H&E staining. **a** Normal, no inflammatory cell infiltration around the airway; **b** asthma, inflammatory cell infiltration around the airway and blood vessels, the airway smooth muscle became thick, subepithelial collagen deposition and fibrosis could be observed; **c**–**f** HDAC inhibitor groups and betamethasone group, inflammatory cell infiltration around the airway and blood vessels was reduced dramatically

In the asthma group, inflammatory cell (eosinophils and neutrophils) infiltration was observed surrounding the peribronchial and perivascular areas and blood vessels, the airway epithelium was exfoliated, and the airway smooth muscle and alveolar septum was thickened. Pathological examination did not show any differences compared with normal mice. In the dexamethasone and HDAC inhibitor group the inflammatory cell infiltration around the airway was reduced compared with the asthma group (Fig. 2).

**Fig. 3:**
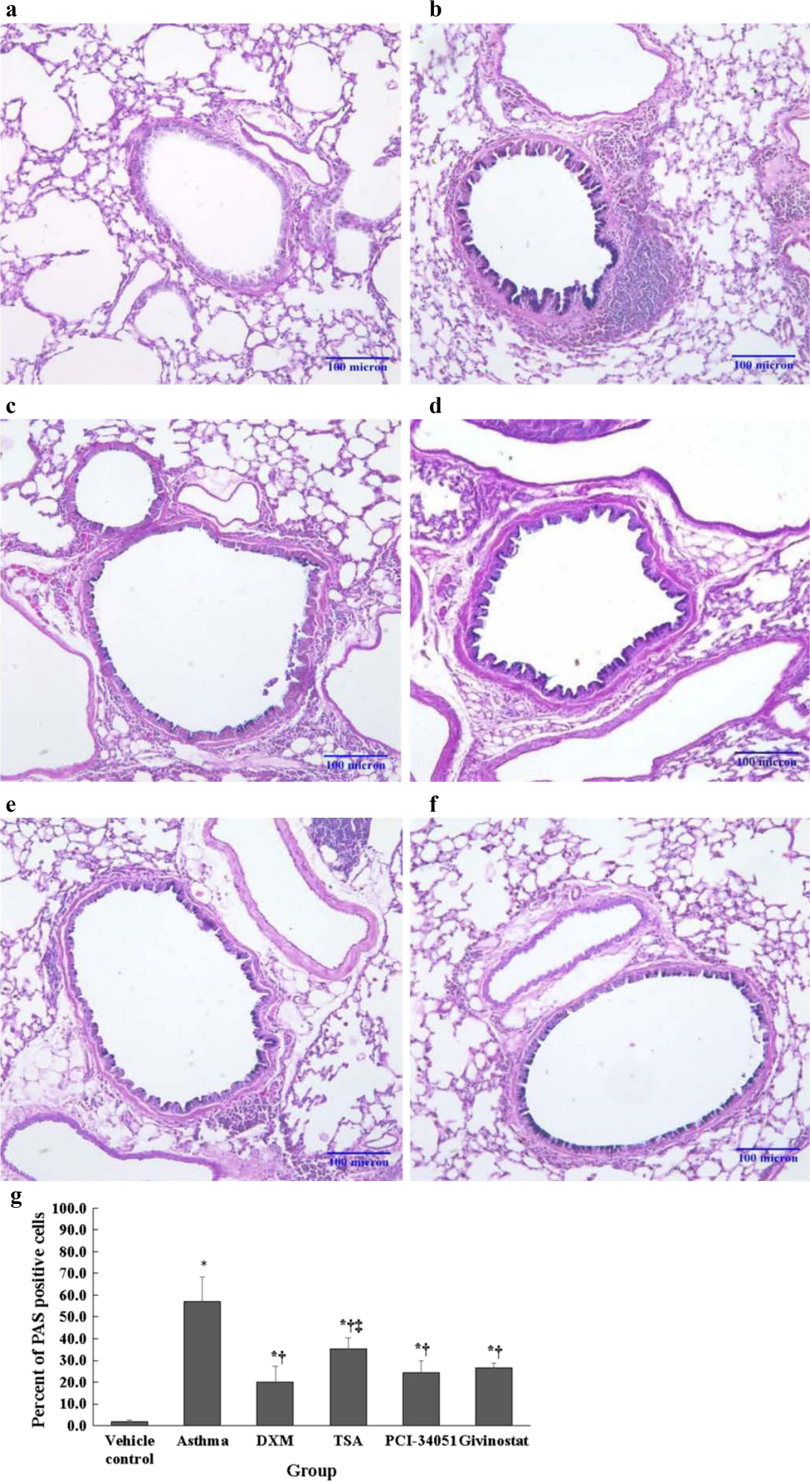
PAS positive cells in airway epithelium. Representative AB-PAS staining images (**a** normal control, **b** asthma, **c** dexamethasone, **d** TSA, **e** PCI-34051, and **f** givinostat). The percent of PAS positive cells (**g**) are presented as mean ± SD for each group (*n* = 6 for each group). Data were compared using a one-way ANOVA with a post hoc pair-wise comparison, Bonferroni test. *p* < 0.05, significantly different compared with *normal control group, ^†^asthma group, or ^‡^DXM group

A higher proportion of PAS positive cells was observed in the asthma, DXM, TSA, PCI-34051, and givinostat groups compared with the normal control group (all *p* ≤ 0.015; Fig. 3). Moreover, the percentage of PAS positive cells was decreased significantly in the DXM, TSA, PCI-34051, and givinostat groups compared with the asthma group (all *p* ≤ 0.033). The TSA had a significantly higher proportion of PAS positive cells compared with the DXM group (*p* = 0.029). There were no significant differences between TSA, PCI-34051, and givinostat groups (Fig. 3g).

**Fig. 4:**
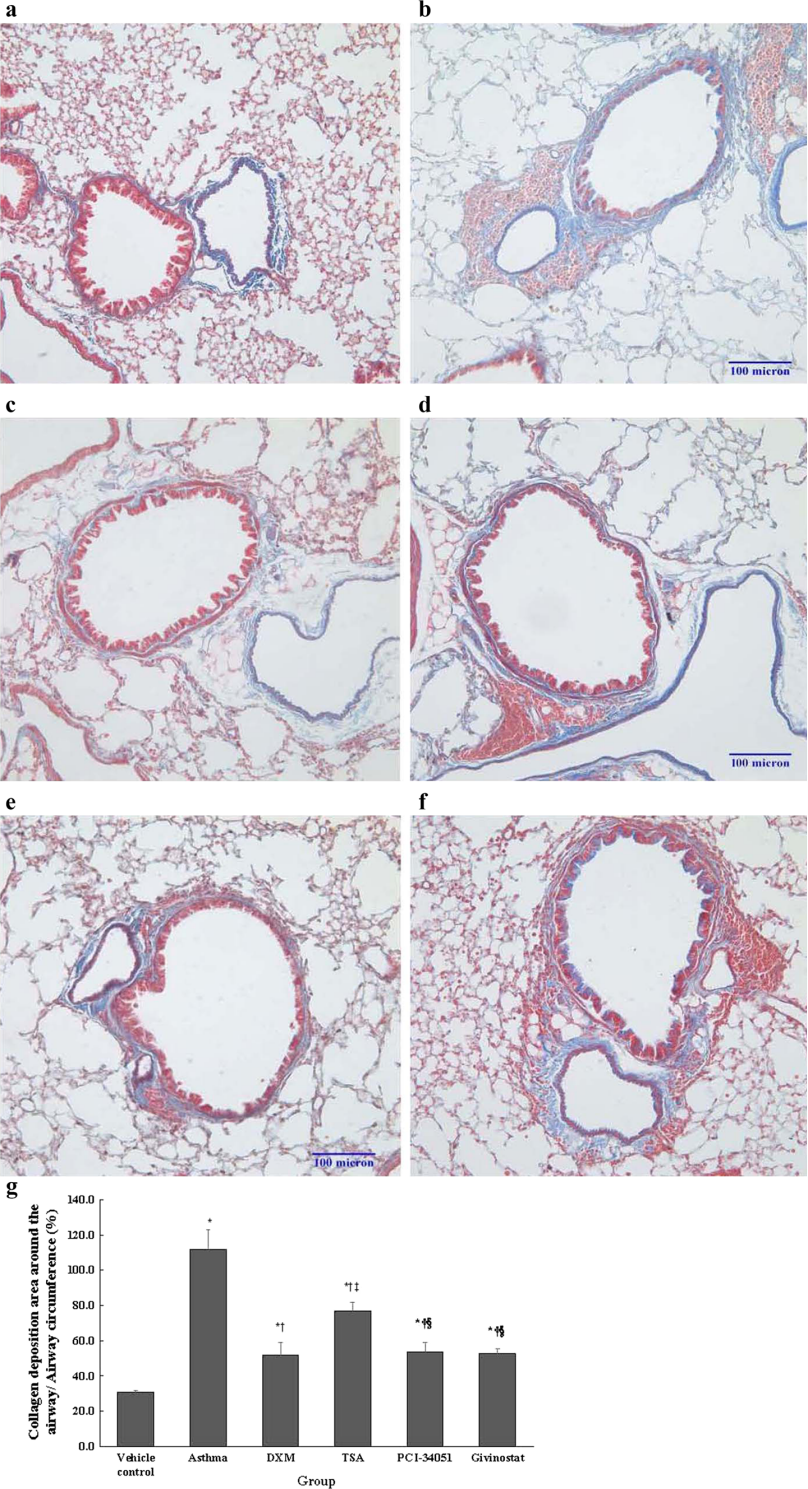
Ratio of collagen deposition around the airway/airway circumference (Masson staining). Representative Masson staining images (**a** normal control, **b** asthma, **c** dexamethasone, **d** TSA, **e** PCI-34051, and **f** givinostat). The percent collagen deposition area (**g**) is presented as mean ± SD for each group (*n* = 6 for each group). The Masson staining positive areas among groups were compared using a one-way ANOVA with a post hoc pair-wise comparison, Bonferroni test. *p* < 0.05, significantly different compared with *normal control group, ^†^asthma group, ^‡^DXM group, or ^§^TSA group

A higher ratio of collagen deposition around the airway was observed in the asthma, DXM, TSA, PCI-34051, and givinostat groups compared with the normal control group (all *p* ≤ 0.010; Fig. 4). Moreover, the ratio of collagen deposition area around the airway was significantly decreased in DXM, TSA, PCI-34051, and givinostat groups compared with the asthma group (all *p* < 0.001). The TSA group had a higher ratio of collagen deposition around the airway compared with the DXM (*p* = 0.001), PCI-34051 (*p* = 0.003), and givinostat (*p* = 0.002) groups, respectively (Fig. 4g).

**Fig. 5:**
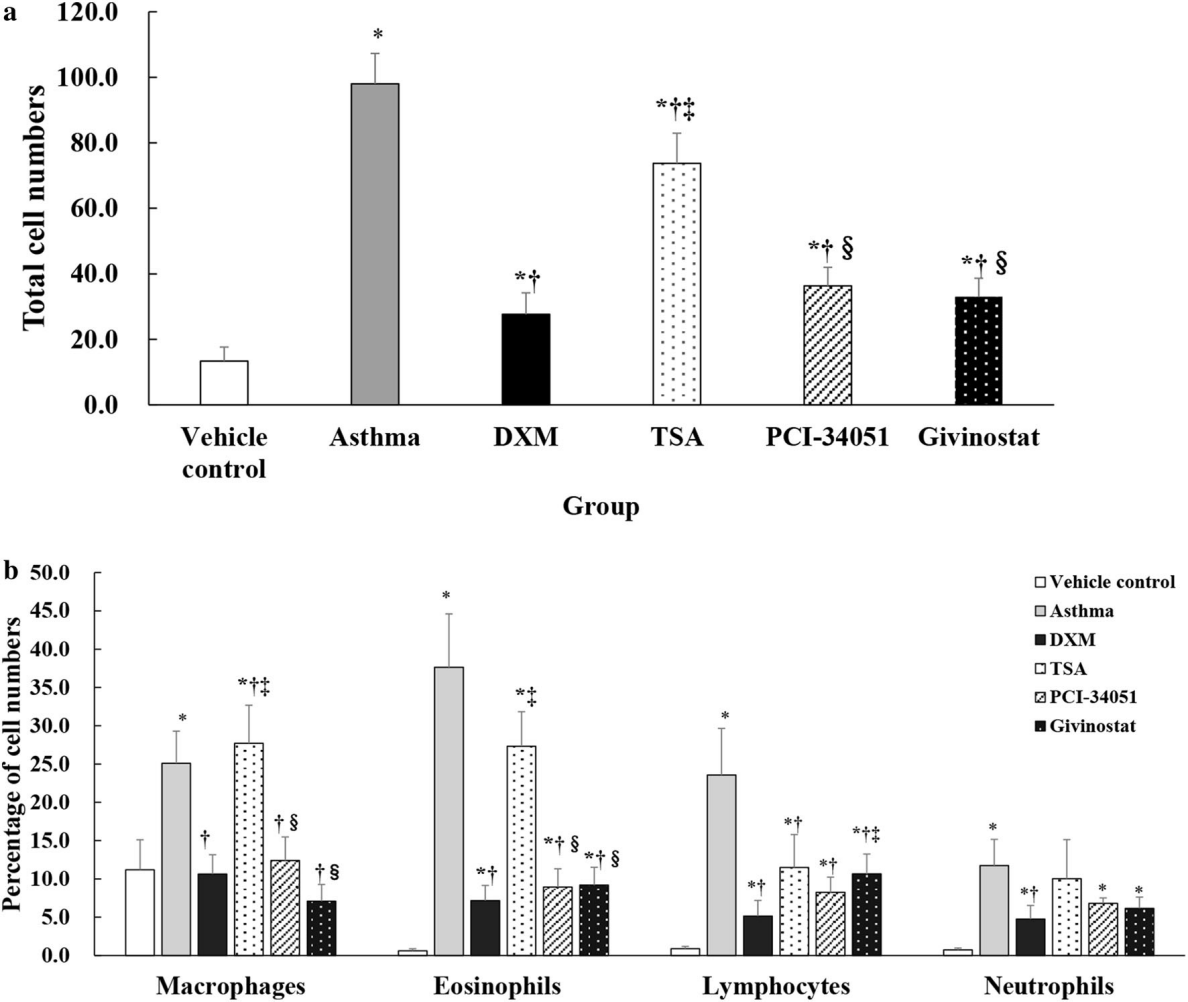
Comparison of total cell number (**a**) and percentages of various cell types (**b**) in the BALF sample (*n* = 6). Data are presented as mean ± SD for each group (*n* = 6 for each group). The cell numbers among groups were compared using a one-way ANOVA with a post hoc pair-wise comparison, Bonferroni test. *p* < 0.05, significantly different as compared to *normal control group, ^†^asthma group, ^‡^DXM group, ^§^TSA group, or ^¶^PCI-34051 group

Total cell numbers were significantly lower in the normal control group compared with other groups (all *p* ≤ 0.020; Fig. 5a). The asthma group had significantly higher total cell numbers than the DXM, TSA, PCI-34051, and givinostat groups (all *p* < 0.001). The percentage of macrophages, and eosinophils in the BALF samples were significantly higher in the TSA group compared with the DXA, PCI-34051, and givinostat groups (all *p* < 0.001; Fig. 5b).

**Fig. 6:**
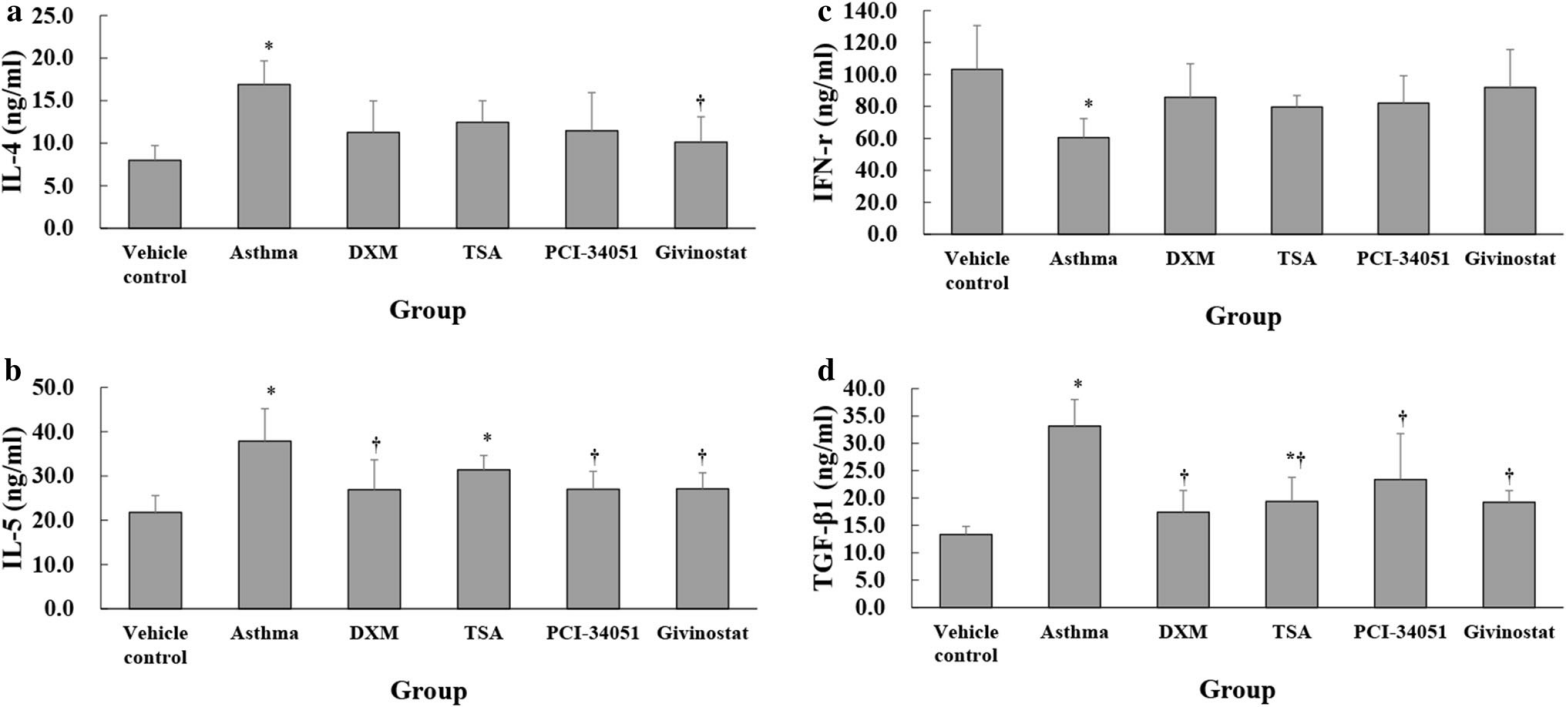
The protein levels of IL-4 (**a**), IL-5 (**b**), IFN-γ (**c**) and TGF-β1 (**d**) in the BALF samples. Data were presented as mean ± SD for each group (*n* = 6 for each group). Differences of among groups for each of outcomes were compared using one-way ANOVA with a post hoc pair-wise comparison, Bonferroni test. *p* < 0.05, significantly different as compared with *normal control group or ^†^asthma group for each of outcomes

The asthma group had significantly higher IL-4 levels compared with the normal control group (*p* < 0.001) and givinostat group (*p* = 0.012; Fig. 6a). The normal control group had significantly lower IL-5 levels compared with the asthma (*p* < 0.001) and TSA groups (*p* = 0.039); whereas, the asthma group had significantly higher IL-5 levels compared with the DXA, PCI-34051, and givinostat groups (all *p* ≤ 0.014; Fig. 6b). The asthma group had significantly lower IFN-γ levels compared with normal control group (*p* = 0.009; Fig. 6c). The normal control group had significantly lower TGF-β1 levels compared with the asthma (*p* < 0.001) and PCI-34051 groups (*p* = 0.014; Fig. 6d). The asthma group had significantly higher TGF-β1 levels compared with the DXM, TSA, PCI-34051, and givinostat groups (*p* ≤ 0.019). The levels of IL-4, IL-5, IFN-γ, and TGF-β1 in the BALF samples in TSA, PCI-34051, and givinostat groups were comparable (all *p* > 0.05).

**Fig. 7:**
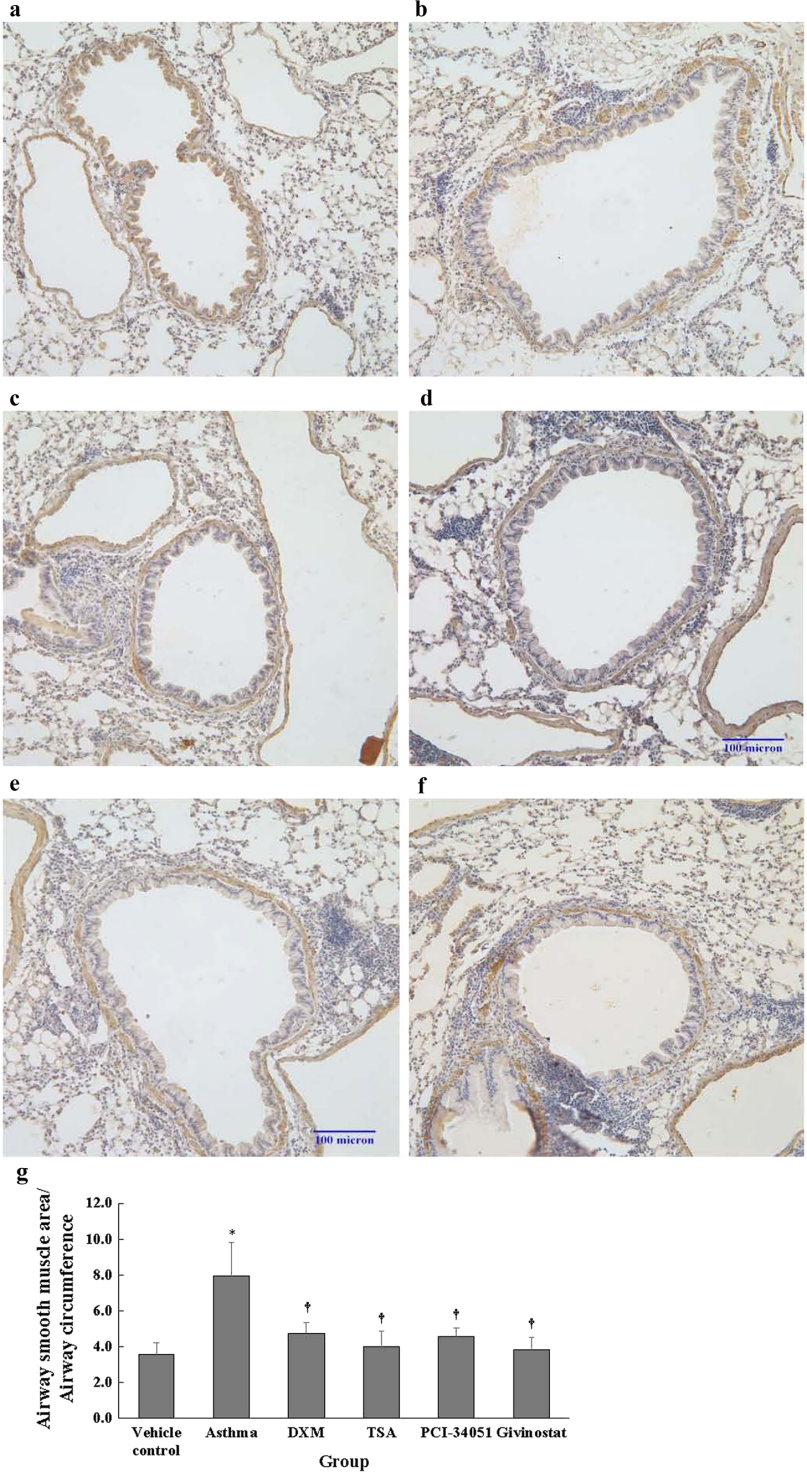
α-SMA immunoreactivity (ratio of airway smooth muscle area/airway circumference). Representative immunohistochemical images (**a** normal control, **b** asthma, **c** dexamethasone, **d** TSA, **e** PCI-34051, and **f** givinostat). Airway smooth muscle area/airway circumference (**g**) are presented as mean ± SD for each group (*n* = 6 for each group). Between group differences were compared using a one-way ANOVA with a post hoc pair-wise comparison, Bonferroni test. *p* < 0.05, significantly different as compared with *normal control group and ^†^asthma group

**Fig. 8:**
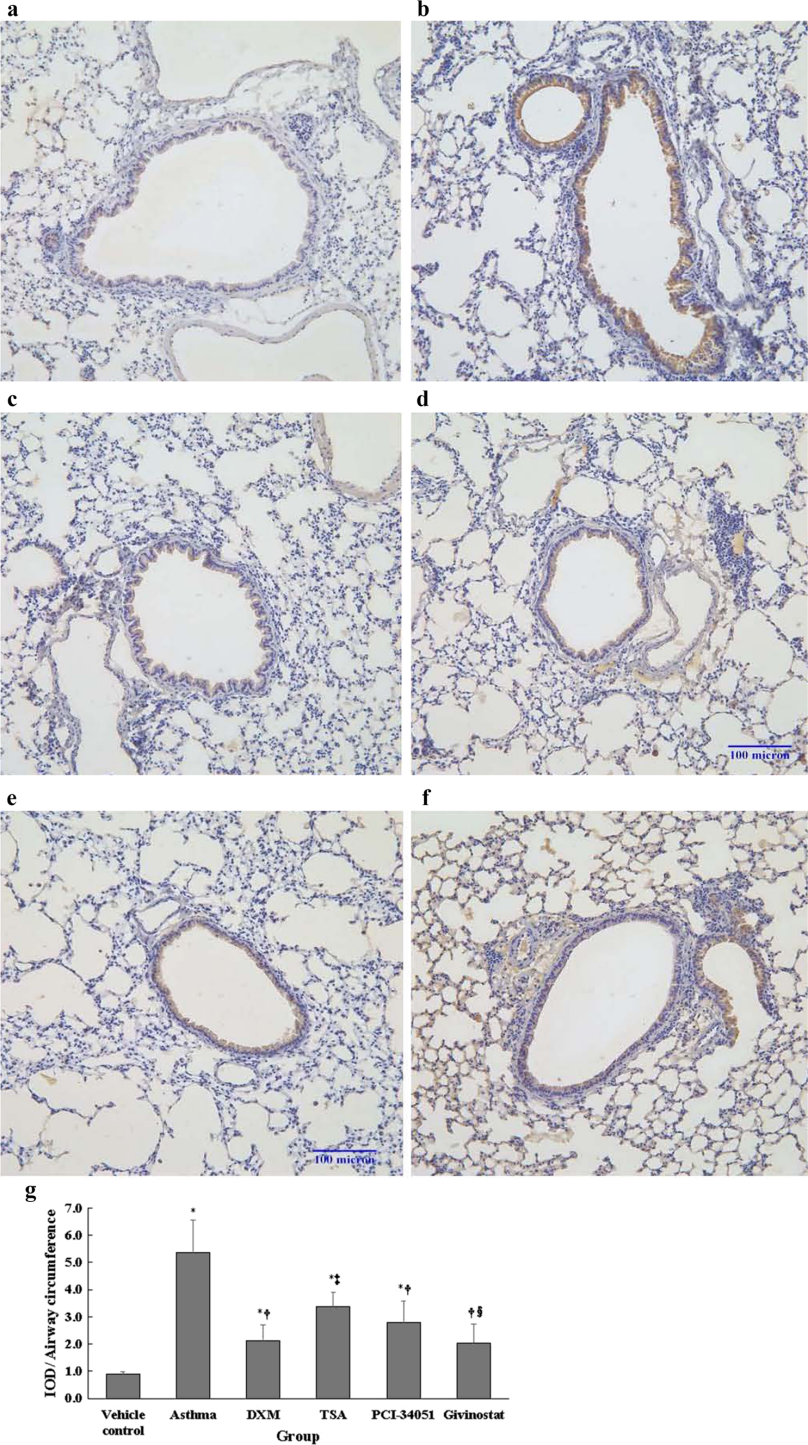
TGF-β1 immunoreactivity [ratio of integrated optical density (IOD)/airway circumference]. The representative immunohistochemical images (**a** normal control, **b** asthma, **c** dexamethasone, **d** TSA, **e** PCI-34051, and **f** givinostat). IOD/airway circumference (**g**) are presented as mean ± SD for each group (*n* = 6 for each group). Between group differences were compared using a one-way ANOVA with a post hoc pair-wise comparison, Bonferroni test. *p* < 0.05, significantly different compared with *normal control group, ^†^asthma group, ^‡^DXM group, and ^§^TSA group

The asthma group had significantly higher α-SMA expression (Fig. 7) compared with the normal control group and other groups (all *p* < 0.001). The normal control group had significantly lower expression of TGF-β1 (Fig. 8) compared with each group, except the givinostat group (all *p* ≤ 0.025). The asthma group had significantly higher TGF-β1 expression compared with DXM, PCI-34051, and givinostat groups (all *p* ≤ 0.022). The TSA group had significantly higher TGF-β1 expression compared with the than givinostat group (all *p* = 0.049).

**Fig. 9:**
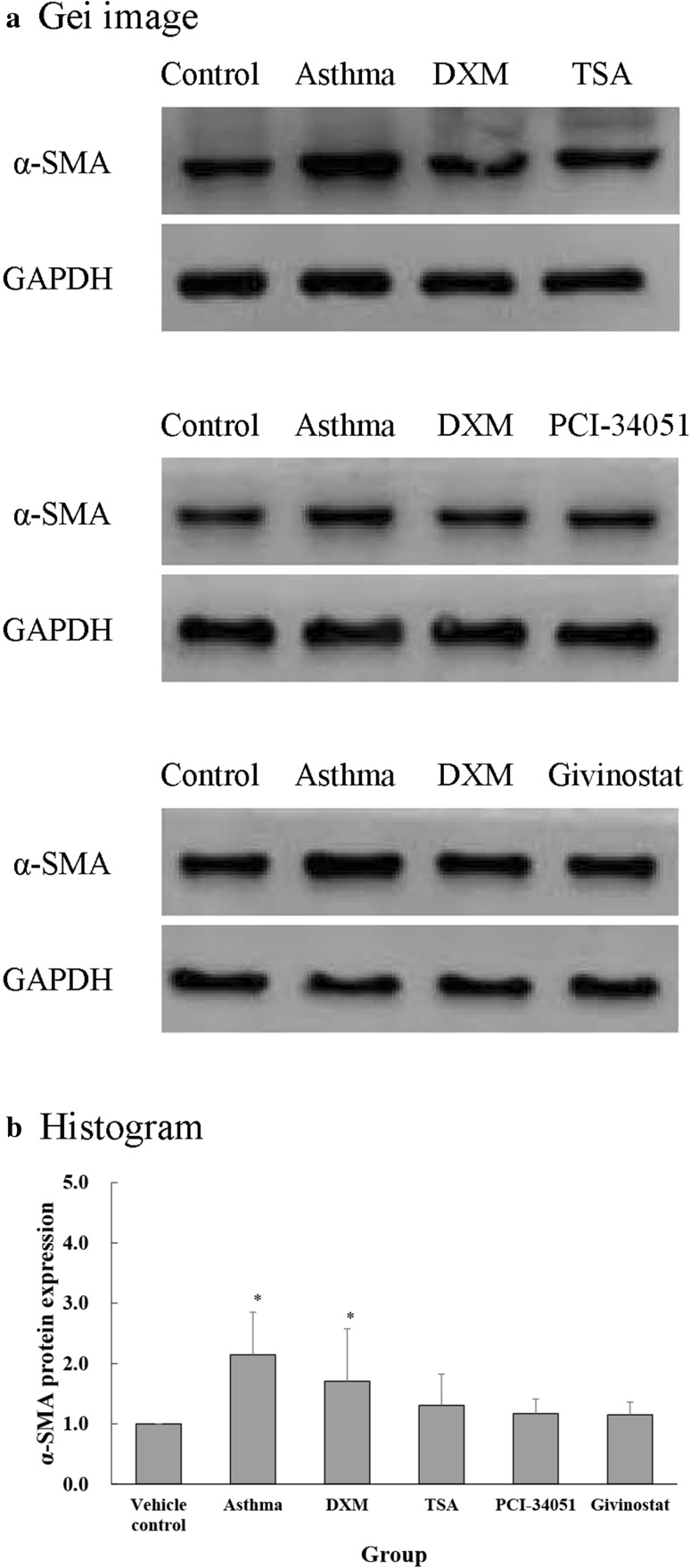
α-SMA protein expression evidenced by western blot. **a** Representative gel images and, **b** α-SMA protein expression presented as mean ± SD for each group. One-sample test was performed the expression as compared to normal control group (setting mean = 1). The between-group differences were compared using a one-way ANOVA with a post hoc pair-wise comparison, Bonferroni test. *p* < 0.05, significantly different as compared with *normal control group or ^†^asthma group

**Fig. 10:**
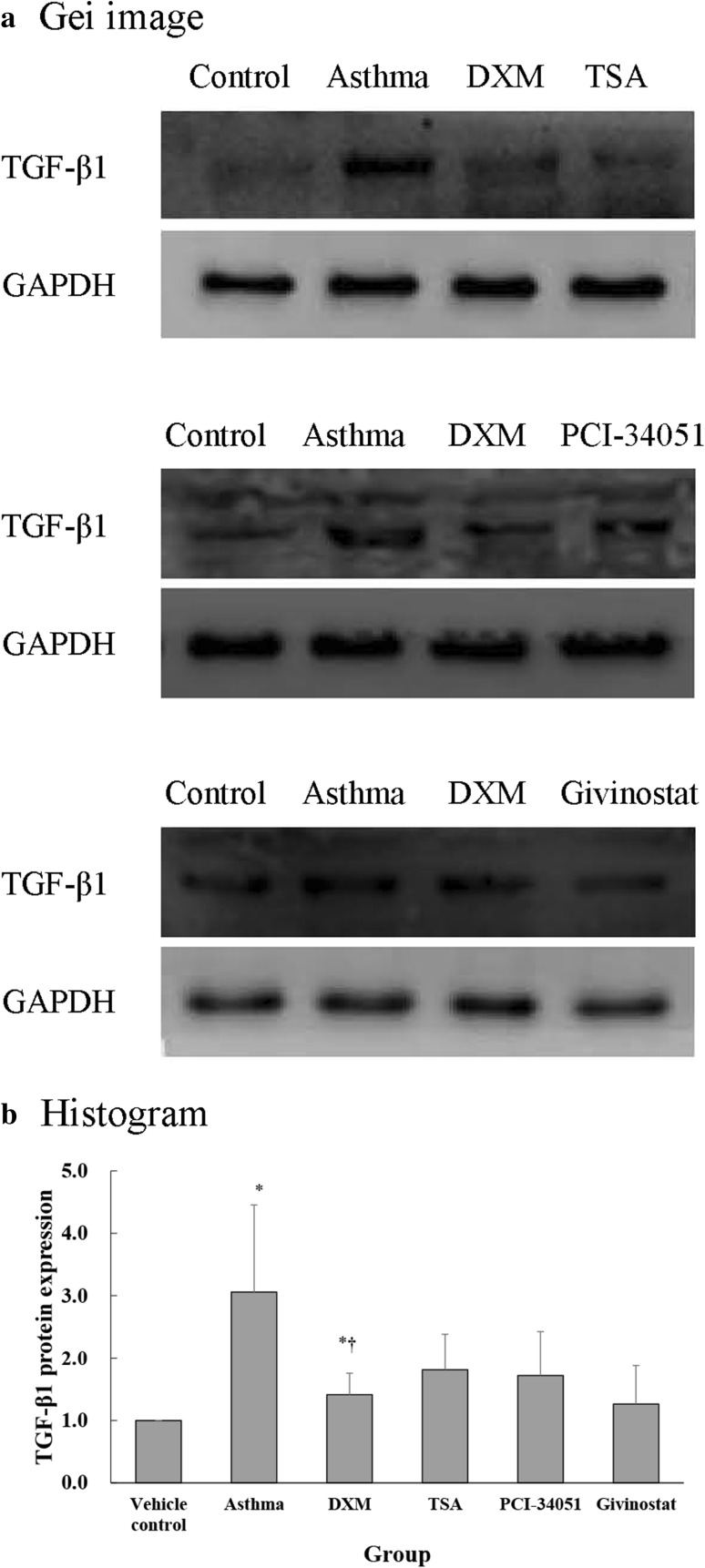
TGF-β1 protein expression evidenced by western blot. **a** Representative gel images and, **b** TGF-β1 protein expression presented as mean ± SD for each group. One-sample test was performed the expression as compared to normal control group (setting mean = 1). The between-group differences were compared using a one-way ANOVA with a post hoc pair-wise comparison, Bonferroni test. *p* < 0.05, significantly different as compared with *normal control group or ^†^asthma group

Protein expression of α-SMA (Fig. 9) and TGF-β1 (Fig. 10) in mouse lungs varied per administered treatments. The asthma and DXM groups had significantly higher α-SMA and TGF-β1 protein expression compared with the normal control group (all *p* ≤ 0.022). The DXM group had significantly lower TGF-β1 protein expression compared with the asthma group (*p* = 0.039).

## Discussion

Airway remodeling, as indicated by significant airway obstruction, smooth muscle hyperplasia, and glandular hypertrophy, can be observed in patients with chronic asthma. Smooth muscle hyperplasia, the primary pathology, aggravates airway hyperresponsiveness, increases airway thickness, and results in irreversible airflow restriction. Furthermore, the secretion of cytokines and extracellular matrix further accelerates airway remodeling [18].

The current investigation used a chronic asthma mouse model to produce airway inflammation, airway remodeling, and airway hyperresponsiveness. Administration of PCI-34051 and dexamethasone reduced the eosinophilic inflammation and airway hyperresponsiveness in asthma to reduce the airway remodeling. Treatment with Tubastatin A HCl reduced airway inflammation and was associated with decreased IL-4, IL-5, and total inflammatory cell count, as well as goblet cell metaplasia and subepithelial fibrosis; however, this outcome was not as effective as that observed with dexamethasone. TGF-β1 expression in the cytoplasm of airway epithelium of mice in the Tubastatin A HCl group was reduced and expression of α-SMA in the airway smooth muscle was also decreased. During our previous experiments, we proved that application of PCI-34051, an inhibitor of HDAC8, could relieve the airway inflammation in asthma and the outcome was as effective as the steroid.

As the most commonly seen member of the histone deacetylase family, HDAC6 is mainly distributed in the cytoplasm and regulates cellular morphology, adhesion, and migration with α-tubulin, HSP90 and cortical actin as the substrates [19], while the cellular migration is a prerequisite of many physiological processes, such as exudation of leukocytes during inflammation, metastasis of cancer cells, wound healing [20, 21]. Worth noting, Ito et al. reported that the expression patterns of HADC1 to HDAC6 in the airway were similar in between normal subjects and subjects with asthma, suggesting that HDAC6 may not play a role in the pathogenesis of asthma [22], possibly as a mediator of LPS-induced macrophage activation [23].

PCI-34051 is a low molecular-weight hydroxamic acid and a specific inhibitor of HDAC8 [12, 24]. In the present study, we found that PCI-34051 could relieve the eosinophilic inflammation and airway hyperresponsiveness in asthma to reduce the airway remodeling. After PCI-34051 administration, we found that the amount of inflammatory cells and EOS cells in the BALF decreased, TH2 cell factor IL-4, IL-5 and TGF-β level reduced significantly, and the inflammatory cell infiltration around the bronchus decreased obviously, which suggests that the PCI-34051 treatment can relieve the TH2 immune response. Improvement of these factors shows that PCI-34051 can effectively improve the level of inflammation in asthma.

Despite recent studies which demonstrated that broad-spectrum histone deacetylase inhibitors can relieve airway inflammation, airway remodeling, and airway hyperresponsiveness associated with asthma [19–22, 25, 26], the relationship between various members of histone deacetylase family and the asthma remains to be fully elucidated. Banerjee et al. reported that the inhibition of HDAC by TSA abrogates airway hyperresponsiveness to methacholine in both naive and antigen-challenged mice; however, they also found that TSA did not affect inflammation. The authors concluded that HDAC inhibitors demonstrate a mechanism of action distinct from that of anti-inflammatory agents such as steroids, and represent a promising therapeutic agent for airway disease [3]. Accumulating evidence, including the current investigation, has indicated beneficial effects in rodent models of allergic airways disease, the potential use of histone deacetylase inhibitors in asthma remains controversial given their mechanisms of action. In short, more specific compounds may have the potential to ameliorate unwanted side effects, but will also help further define HDAC inhibitors mechanism of action in asthma [27].

The results suggested that treatment with HDAC inhibitors can reduce airway inflammation, airway remodeling, and airway hyperresponsiveness in a mouse chronic allergic airway disease model. Occurrence of airway remodeling in asthma is related to the persistent airway inflammation and repeated airway epithelium damage/repair. It has been verified that HDAC6 together with HDAC4 and HDAC8 regulates TGF-β1-mediated α-SMA expression in the smooth muscle and takes part in the maintenance of cellular morphology and tissue repair [25]. During the airway remodeling in asthma, Tubastatin A HCl can inhibit the TGF-β1 expression and further restrict the airway epithelial-mesenchymal transition to relieve the airway remodeling in asthma. It was reported that HDAC6 inhibitors might be involved in the biomechanical behaviors of the airway smooth muscle cells, such as contraction of the airway smooth muscle cells, fibrous structure of the cytoskeleton and tension of the airway smooth muscle cells [8, 12, 26, 28] to affect the proliferation, migration and differentiation of airway smooth muscle cells and restrict the development of airway remodeling. In the present study, we found that HDAC6 could relieve the airway inflammation, airway remodeling and airway hyperresponsiveness in asthma. However, the role of HDAC6 in the treatment of asthma may not be realized by conventional anti-inflammatory effects but achieved by relieving the epithelial damage/repair and proliferation, differentiation and migration of the airway smooth muscle. Further studies are planned to help further clarify the proposed therapeutic pathways.

### Study limitations

In this study, a chronic asthma model was employed to investigate the therapeutic effects of different HDAC inhibitors on airway inflammation, airway remodeling, and airway hyper responsiveness. Studies revealed that a HDAC8 specific inhibitor (PCI-34051) had advantages in the relief of airway inflammation associated with asthma. Furthermore, the inhibitory effects of another HDAC inhibitor (Tubastatin A HCl) on airway inflammation was slightly inferior to other drugs, but the protective effects of Tubastatin A HCl on airway hyper responsiveness and airway remodeling (especially on the expression of TGF-β1 and α-SMA) were comparable to those of other drugs. Thus, we speculate that a HDAC8 specific inhibitor may relieve airway inflammation, airway remodeling, and airway hypersensitivity via its anti-inflammatory effect; it may also inhibit the persistent inflammation in the airway to relieve the airway remodeling associated with asthma.

An HDAC6 specific inhibitor, however, is more likely to inhibit the remodeling of structural cells in the airway thereby inhibiting airway remodeling and airway hypersensitivity. Worth noting, HDAC6 specific inhibitors affect some biological activities of cells such as exudation, migration and aggregation of inflammation, suggesting the anti-inflammatory effect of this drug, but its anti-inflammatory effect is not as potent as that of other anti-inflammatory drugs such as steroids. We therefore speculate that HDAC6 specific inhibitors may have limitations in the treatment of acute inflammation, which may not be evident in the model of chronic inflammation currently described. We propose an early acute asthma model would be better suited for examining the effects of HDAC inhibitors on inflammation observed in asthma.

Future studies will employ an early acute asthma model to confirm the hypothesis that specific HDAC8 inhibitors may exert anti-inflammatory effects and the anti-inflammatory, anti-remodeling and anti-hypersensitivity effects are closely related to potent anti-inflammatory activity in asthma.
